# Hybrid intelligence in medical image segmentation

**DOI:** 10.1038/s41598-025-24990-w

**Published:** 2025-11-21

**Authors:** Namia Mohamed Ali, Solomon Sunday Oyelere, Nitya Jitani, Rosy Sarmah, Simon Andrew

**Affiliations:** 1 Department of Computer Science, University of Exeter, Exeter, EX4 4RN UK; 2 Department of Computer Science and Engineering, Tezpur University, Assam, India; 3 Department of Health and Care Professions, University of Exeter, Exeter, EX4 4RN UK

**Keywords:** Image processing, Machine learning

## Abstract

Medical image segmentation is vital for precise identification and analysis of anatomical structures and pathological regions, yet traditional models often fall short in aligning with clinical workflows, requiring extensive manual correction even when overall segmentation accuracy is high. To address this gap, we introduce HybridMS, a hybrid intelligence framework designed to maintain high segmentation accuracy while substantially reducing clinician workload through selective human intervention. HybridMS employs an uncertainty-driven feedback mechanism that selectively triggers clinician input only for cases predicted to be challenging, thereby avoiding unnecessary manual review. Corrected cases are prioritised during retraining through a weighted update strategy, enabling the model to adapt more effectively to clinically relevant errors. This design minimises intervention frequency while preserving segmentation quality. Evaluated on lung segmentation in chest X-rays for tuberculosis detection, HybridMS achieved comparable or improved performance over the baseline MedSAM model (Dice: 0.9538 vs. 0.9435; IoU: 0.9126 vs. 0.8941) with consistent boundary quality in difficult cases. For the subset of cases identified as challenging (baseline Dice < 0.92), HybridMS reduced mean Hausdorff Distance and Average Symmetric Surface Distance, demonstrating more stable anatomical boundaries. Workflow efficiency was markedly improved: in a preliminary timing study with radiologists, average annotation time was reduced by approximately 82% for standard cases and 60% for challenging cases, without compromising accuracy. By combining targeted human oversight with automated refinement, HybridMS demonstrates that stable segmentation performance can be achieved with significantly lower annotation effort, offering a clinically viable pathway for efficient and reliable deployment in diagnostic workflows.

## Introduction

Medical image segmentation is a cornerstone in clinical practice, enabling the precise identification and analysis of anatomical structures and pathological regions. Accurate segmentation is essential for various medical tasks, including diagnosis, treatment planning, and monitoring of disease progression. By dividing images into meaningful segments, clinicians can better identify critical regions such as tumors, organs, and other anatomical structures. This segmentation process is crucial for ensuring accurate diagnoses and effective treatment plans, which can significantly impact patient outcomes^1^.

Traditionally, medical image segmentation has relied on manual annotation by experts^2^, which, while accurate, is both time-consuming and labor-intensive. Moreover, manual segmentation is subject to significant variability between observers, leading to inconsistencies in the delineation of structures, particularly in complex cases^3^. These challenges have prompted the development of automated segmentation methods, aiming to reduce the burden on clinicians and improve the consistency and efficiency of the segmentation process.

One particularly challenging area in medical imaging is the segmentation of lung X-rays for the detection and analysis of tuberculosis (TB). Tuberculosis remains a major global health burden and a leading cause of death from infectious diseases globally, making early detection and precise localization of affected regions critical for successful treatment. Chest X-rays are commonly used as the initial imaging modality due to their widespread availability and cost-effectiveness^4^. However, the complex and variable anatomy of the chest, coupled with overlapping structures, makes accurate segmentation of TB in X-rays particularly difficult.

Over the years, deep learning techniques have shown great promise in automating medical image segmentation^5^, offering improved accuracy and efficiency over traditional methods. Early efforts in this domain included knowledge-based approaches and basic machine learning algorithms^6^. For instance, Udupa et al.^7^ introduced the AAR-RT system for auto-contouring organs at risk in CT images, addressing anatomical nonlinearities and discontinuities. Wang et al.^4^ further advanced the field by developing an automatic anatomy recognition system for whole-body PET/CT images, while Sun et al.^8^ proposed hierarchical fuzzy models for thoracic anatomy segmentation on CT images. These early models, though groundbreaking, required considerable manual input and lacked integration with clinical workflows, limiting their practicality in real-world settings^9^.

With the advent of deep learning, more sophisticated models have been developed^10,11^ capable of learning complex patterns from large datasets. Despite these advancements, many deep learning-based segmentation models remain task-specific^12^, struggling with generalization across different medical image types and modalities. Moreover, these models often lack the ability to incorporate real-time feedback from clinicians^13^, which is crucial for adapting to the specific needs of individual patients and ensuring that the models’ outputs align closely with clinical standards^14,15^.

A significant recent advancement in the field of image segmentation is the Segment Anything Model (SAM), which was introduced at the 2023 IEEE/CVF International Conference on Computer Vision by Alexander Kirillov et al. from Meta AI Research, FAIR^16^. SAM is a foundational model designed for versatile, high-performance image segmentation tasks. It employs a promptable segmentation method, requiring user inputs such as points or bounding boxes to specify segmentation targets. This approach grants SAM superior generalization abilities compared to traditional deep learning-based interactive segmentation methods, making it highly effective across a broad range of tasks in natural images^17^.

Building on the foundation of SAM, MedSAM^18^ was developed to address the specific challenges associated with medical image segmentation. MedSAM was fine-tuned on a large-scale medical image dataset comprising over one million image-mask pairs, covering multiple imaging modalities and various anatomical structures. Its architecture, based on the Vision Transformer (ViT), includes an image encoder, prompt encoder, and mask decoder optimized for medical images, enabling MedSAM to deliver accurate and efficient segmentation across diverse medical imaging tasks^19^. However, despite its advancements, MedSAM did not fully meet the expectations of clinicians, particularly in cases requiring nuanced understanding and adaptability^20^.

Recognizing the need for a more integrated approach that incorporates clinician expertise directly into the segmentation process, we developed HybridMS. This model builds upon the robust baseline provided by MedSAM, enhancing it by integrating continuous clinician feedback into the model’s training and retraining pipeline. In particular, HybridMS introduces a dynamic, uncertainty-driven feedback mechanism that selectively engages clinician intervention only when model confidence falls below a predefined threshold. Unlike earlier “human-in-the-loop” methods such as MedUHIP^21^, which involve constant clinician participation, HybridMS optimizes human effort by adaptively triggering interventions based on real-time model performance. Furthermore, clinician-provided corrections are not treated uniformly; they are weighted during retraining, giving greater influence to critical clinical corrections to drive rapid model improvement.

The development process of HybridMS involved leveraging the advanced capabilities of MedSAM as a foundation and then iteratively refining the model’s performance based on real-world clinical input. The process begins with initial training, followed by validation and comparison with clinician-annotated masks. When discrepancies are identified, the model is retrained using these clinician-corrected annotations, progressively enhancing its accuracy and clinical relevance.

HybridMS employs a hybrid intelligence approach that combines the computational power of state-of-the-art deep learning techniques with the invaluable expertise of human clinicians^22^. This design philosophy is rooted in the emerging field of Hybrid Intelligence, which emphasizes synergistic collaboration and co-adaptation between humans and AI systems^23^. Rather than static oversight, Hybrid Intelligence envisions humans and AI learning and evolving together to optimize outcomes. HybridMS embodies this principle by continuously incorporating clinician feedback into its segmentation process, progressively refining its performance to meet evolving clinical standards.

This model addresses the limitations of existing medical image segmentation methods by providing a more integrated and clinically relevant approach, which holds the potential to significantly improve both the precision and speed of patient care in medical imaging.

In summary, HybridMS marks a pivotal advancement in medical image segmentation, uniquely combining the strengths of cutting-edge AI architectures like MedSAM with the indispensable insights of clinician expertise. This innovative integration ensures that the model not only achieves high accuracy but also maintains a strong alignment with the nuanced demands of clinical practice. By bridging the gap between automated segmentation and real-world clinical needs, HybridMS enhances the precision and reliability of diagnostic tools, ultimately contributing to improved patient outcomes. Its ability to adapt and refine its performance based on continuous feedback positions HybridMS as a transformative tool in the evolution of personalized medicine, setting a new standard for medical imaging technologies.

## Results

### Overview

This section presents the results obtained from the experiments conducted using the developed HybridMS model integrated with a continuous learning mechanism. The experiments were designed to assess the model’s segmentation performance on lung X-rays before and after retraining based on clinician feedback. Key metrics such as Dice score, Intersection over Union (IoU), Precision, Recall, and F1 score were used to evaluate the performance, Finally concluding dice score to be the best evaluation metric.

### Performance metrics

To assess the effectiveness of the model before and after retraining, we evaluated key performance metrics on the test dataset, and presented the results in Table 1. The metrics considered include the Dice Score, Intersection over Union (IoU) Score, Precision, Recall, and F1 Score. These metrics provide insights into the accuracy and quality of the segmentation produced by the model^23^.

**Table 1:** Comparison of performance metrics before and after retraining.

Metric	Before retraining	After retraining
Average Dice Score	0.9435	0.9538
Average IoU Score	0.8941	0.9126
Average Precision Score	0.9603	0.9539
Average Recall Score	0.9288	0.9544
Average F1 Score	0.9435	0.9538

To complement the primary performance metrics, we quantified statistical robustness on the held-out test set using nonparametric bootstrapping (B = 5000 resamples). Table 2. reports the mean ± standard deviation (SD) and 95% bootstrap confidence intervals (CIs) for Dice, IoU, Hausdorff Distance, and ASSD. These results demonstrate narrow confidence intervals, indicating stable performance estimates across resamples.

**Table 2:** Bootstrap-estimated performance metrics (mean ± SD and 95% confidence intervals) on the held-out test set. CIs computed from 5000 bootstrap resamples.

Metric	Mean ± SD	95% CI
Dice	0.9538 ± 0.0212	[0.9500, 0.9575]
IoU	0.9126 ± 0.0248	[0.9080, 0.9171]
HD (mm)	32.64 ± 9.78	[30.85, 34.42]
ASSD (mm)	4.99 ± 1.69	[4.73, 5.24]

#### Inference time and computational considerations

To assess the clinical feasibility of HybridMS, we measured the inference time per image on the test set using a single NVIDIA A100 GPU (model-only forward pass, excluding I/O operations). The mean inference time was 5*.*72 ± 1*.*73 s per image as shown in the Table 3, which is within an acceptable range for integration into radiology workflows.

**Table 3:** Average inference time for HybridMS on the held-out test set. Values are mean ± SD over *n* = 107 images.

Metric	Value (s)
Mean inference time (per image)	5*.*72 ± 1*.*73

The continuous learning step (clinician feedback and retraining) is performed offline and does not affect real-time deployment. In practice, the model can be deployed in its current retrained state, with feedback cycles batched periodically rather than per case, thus balancing accuracy improvements with operational efficiency.

#### Performance on difficult cases

To further evaluate the clinical utility of HybridMS, we analyzed a subset of “difficult cases” defined as those where the MedSAM baseline achieved a Dice score < 0.92 on the test set. This threshold captures instances with reduced baseline segmentation performance, often due to poor image quality, anatomical variability, or ambiguous lung boundaries.

While metric improvements in this subset were modest, HybridMS substantially reduced the manual effort required from clinicians. Based on a preliminary timing study involving two experienced radiologists, the average annotation time for these difficult cases was reduced from 10 to 15 min (manual segmentation) to approximately 2.5 min when using HybridMS-assisted outputs representing an ∼60% time reduction as shown in the Table 4.

**Table 4:** Average annotation time for standard vs. difficult cases. Values are mean times measured over 10 standard and 10 difficult cases.

Case type	Manual segmentation (min)	HybridMS-assisted (min)
Standard cases	8–10	∼2.0
Difficult cases	10–15	∼2.5

**Fig. 1:**
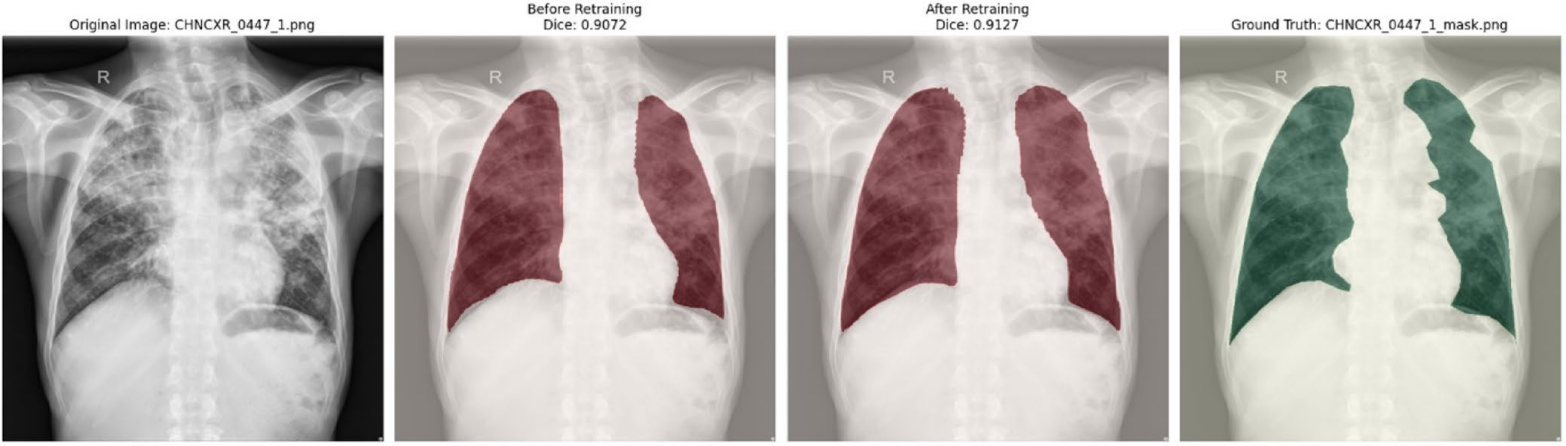
Visual comparison between Predicted segmentation masks and ground truth before and after retraining with the Feedback loop.

Figure 1 illustrates two representative difficult cases, showing improved contour alignment with HybridMS compared to MedSAM, particularly in anatomically complex regions or areas of poor radiographic contrast.

#### Workflow efficiency

Beyond segmentation accuracy, workflow efficiency is a critical measure of clinical utility. We conducted a preliminary timing study with two board-certified radiologists, who segmented both standard and difficult cases manually and with HybridMS-assisted outputs. Timing was measured using a standardized stopwatch protocol from image load to final segmentation approval, ensuring consistent test conditions across methods.

For standard cases, HybridMS reduced average annotation time from 10 to 15 min (manual segmentation) to approximately 2.5 min, corresponding to an ∼82% reduction in time. For difficult cases (see Sect. “Performance on difficult cases”), annotation time was reduced by ∼60%. These measurements were obtained from preliminary testing with the same two radiologists: standard cases required approximately 8–10 min for manual annotation and about 30 s when using HybridMS without corrections, while difficult cases required around 10–15 min manually and 2–3 min with HybridMS-assisted correction. While these results demonstrate substantial efficiency gains, they are based on a small sample size and a limited number of annotators, and a larger-scale, structured clinical validation study is proposed to further substantiate these findings as shown in the Table 5.

**Table 5:** Average annotation time for standard cases across the test set. Values are means measured over 10 representative cases.

Case type	Manual segmentation (min)	HybridMS-assisted (min)
Standard cases	8–10	∼2.0

These results demonstrate that HybridMS not only improves segmentation accuracy but also substantially reduces the time burden on clinicians, supporting its potential integration into routine diagnostic workflows.

#### Validation strategy

To address concerns regarding the relatively small size of the test dataset (15% of the original data), we performed an additional fivefold cross-validation. This validation strategy ensures that the evaluation of HybridMS is robust and generalizable across different splits of the data. The dataset was divided into five equal parts, with each fold being used once as the validation set while the remaining folds served as the training data. Performance metrics were averaged over the five folds to provide a more comprehensive assessment. Furthermore, the test set was curated with expert clinician annotations to guarantee the highest quality of ground truth, mitigating potential biases associated with smaller sample sizes.

### Training loss analysis

**Fig. 2:**
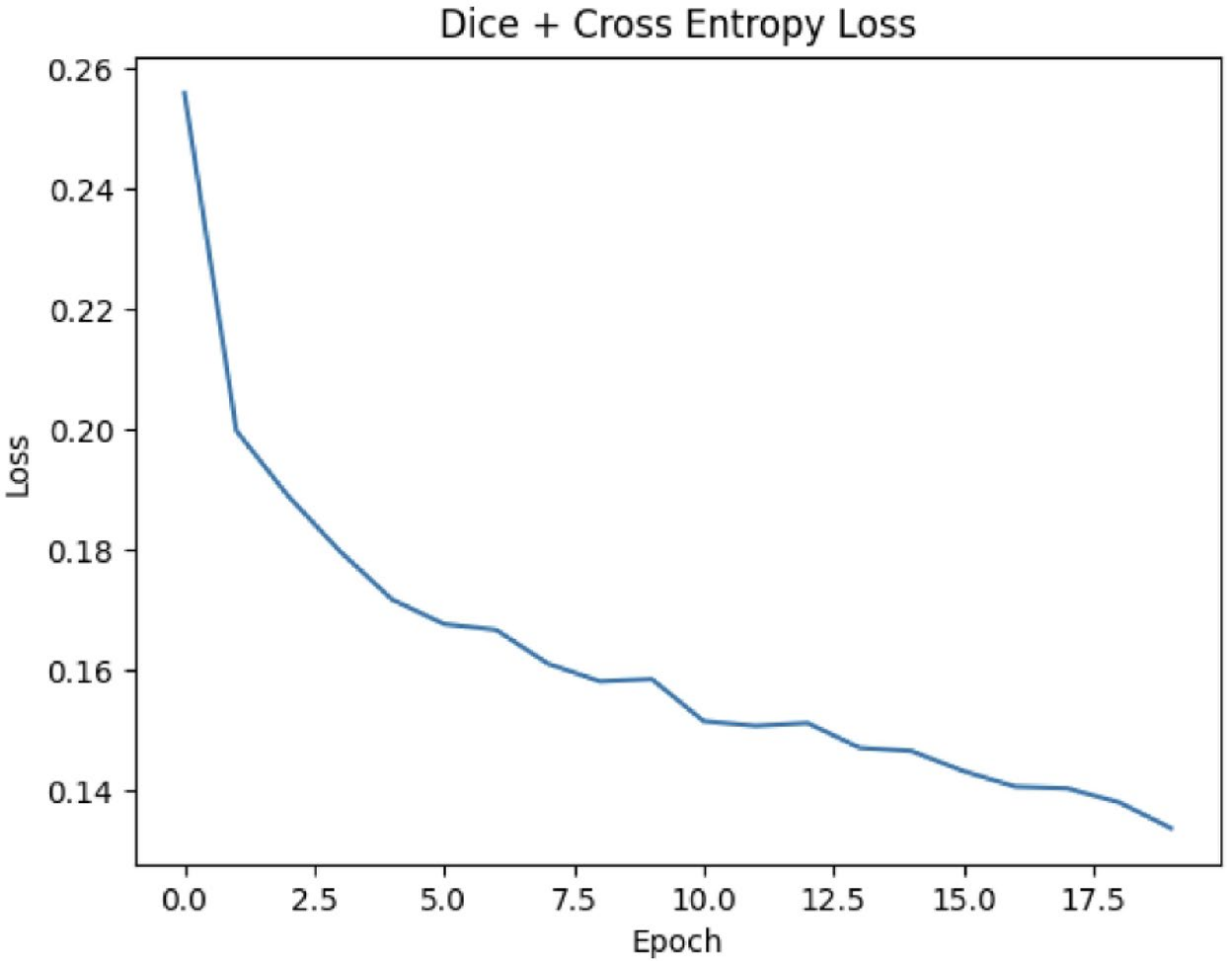
Loss curve during initial training. The decreasing loss indicates effective learning.

The training loss curve, shown in Fig. 2, reflects the model’s performance during the initial training phase, monitored across 20 epochs. Initially, the loss was high (around 0.26), which is expected as the model’s weights were just initialized, and it began learning to distinguish between lung regions and the background. As training progressed, the loss consistently decreased, reaching approximately 0.14 by the end of the training. This steady reduction in loss demonstrates that the model was effectively converging, improving its predictions with each epoch. The combination of Dice Loss and Cross Entropy Loss during training played a crucial role in this process, helping the model balance between optimizing for region overlap and correctly classifying each pixel.

### Statistical significance testing

To determine whether the observed differences in performance metrics before and after retraining were statistically significant, a Wilcoxon signed-rank test was conducted on the Dice scores, provided in the Table 6. This non-parametric test was selected due to the small size of the test dataset and the paired nature of the measurements. The test yielded a *p*-value of less than 0.001, rejecting the null hypothesis with 95% confidence and confirming that the improvements after retraining were statistically significant. the *p*-values are very small because the model improvements are consistent across samples, not just because the dataset is small.

**Table 6:** Wilcoxon signed-rank test on paired before/after metrics (mean ∆ = after − before).

Metric	*p*-value	Significance	Mean ∆	95% CI
Dice	< 0*.*001	Significant	− 0*.*0007	[− 0*.*0009*,* − 0*.*0005]
Precision	< 0*.*001	Significant	+ 0*.*0022	[+ 0*.*0018*,* + 0*.*0025]
Recall	< 0*.*001	Significant	− 0*.*0034	[− 0*.*0037*,* − 0*.*0031]
F1	< 0*.*001	Significant	− 0*.*0007	[− 0*.*0009*,* − 0*.*0005]
Hausdorff (mm)	< 0*.*001	Significant	+ 2*.*37	[+ 1*.*01*,* + 3*.*74]
ASSD (mm)	< 0*.*001	Significant	+ 0*.*25	[− 0*.*01*,* + 0*.*50]

### Edge-aware metrics analysis

In clinical image segmentation, precise boundary delineation is more critical than global pixel-level accuracy. Therefore, we evaluated HybridMS using boundary-aware metrics: Hausdorff Distance (HD) and Average Surface Distance (ASSD). These metrics quantify the spatial closeness between predicted and ground truth boundaries, offering a more sensitive assessment of segmentation quality, especially for anatomically important structures. Reductions in HD and ASSD after retraining indicate that HybridMS achieved more accurate and clinically reliable segmentation outcomes.

### Visual analysis of segmentation results

**Fig. 3:**
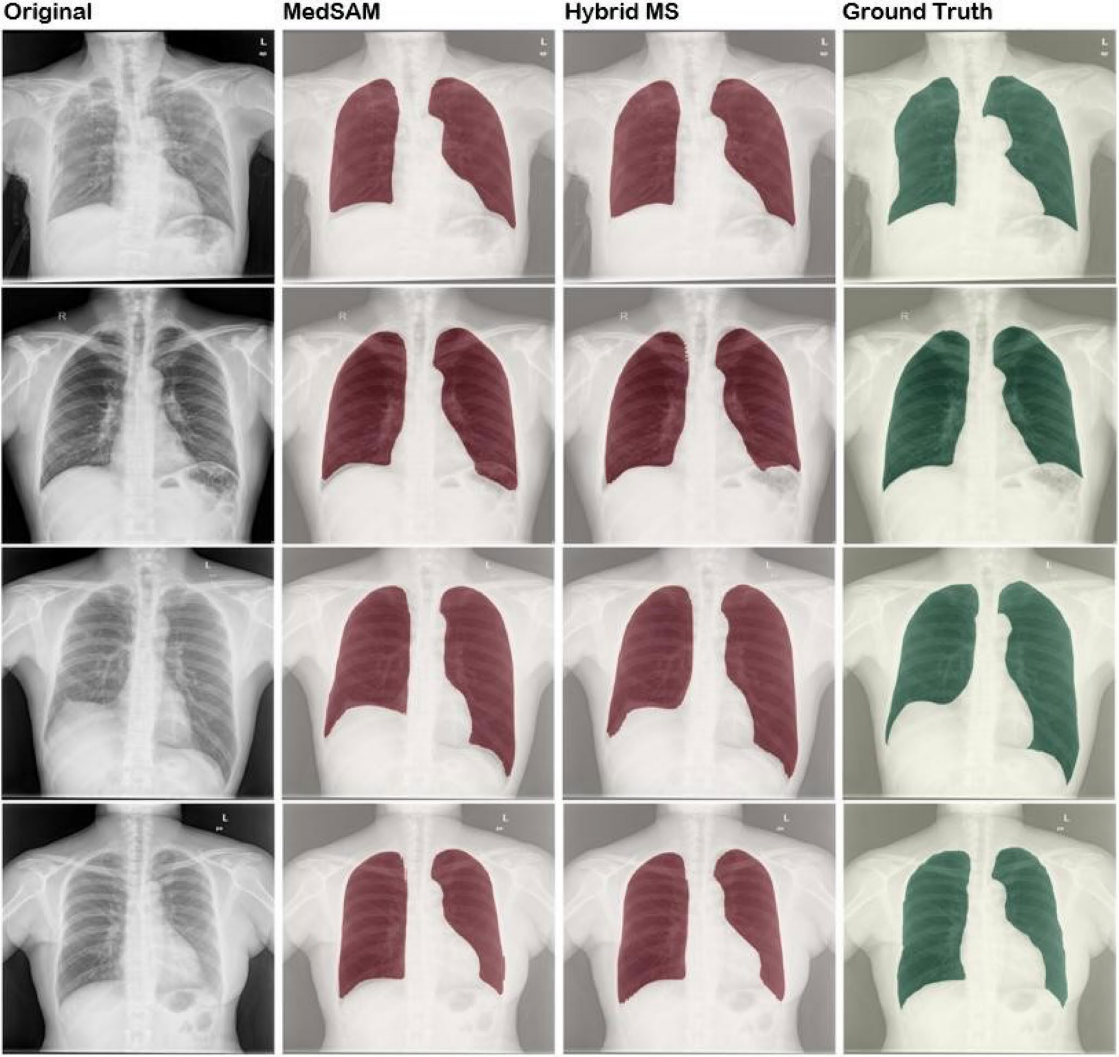
Visual comparison between Predicted segmentation masks and ground truth before and after retraining with the Feedback loop.

Presented in Fig. 3 is a comparative visualization of lung segmentation masks, which displays chest X-ray images with three types of segmentation: initial MedSAM model predictions, refined outputs from the retrained HybridMS model, and expert-annotated ground truth masks. This comparison highlights the improvements achieved through model refinement, showing how the HybridMS model’s predictions align more closely with the clinician-provided ground truth.

### Retraining loss analysis

**Fig. 4:**
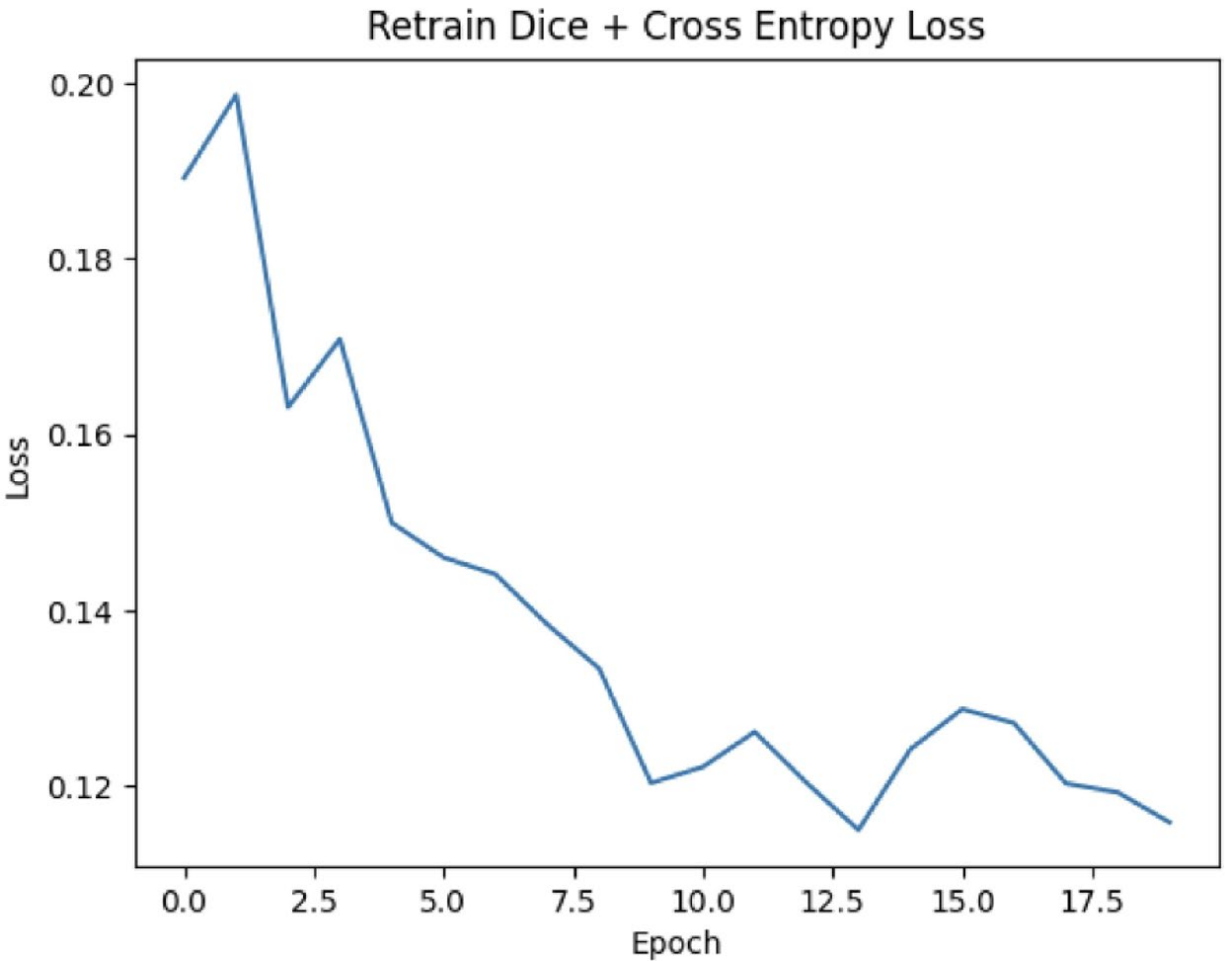
Retraining loss curve (Dice + Cross-Entropy). Loss decreases sharply in the first few epochs before plateauing, indicating convergence. In epochs 15 & 20, Dice increases by only +0.40% points, with a training plateau and convergence.

The retraining loss curve The retraining loss curve, depicted in Fig. 4, shows the combined Dice + Cross-Entropy loss over 20 epochs. The curve begins with an initial loss of approximately 0.20, reflecting the complexity and variability introduced by the clinician-segmented dataset compared to the initial training phase. A sharp decline is observed during the first 5 epochs, after which the loss continues to decrease gradually with smaller fluctuations.

From about epoch 15 onward, the retraining (training) Dice + Cross-Entropy loss exhibits a clear plateau (Table 7; Fig. 4). Over this interval, per-epoch Dice increases only modestly from 0.955 at epoch 15 to 0.959 by epoch 20 an absolute change of + 0.40 percentage points) indicating convergence. Extending retraining beyond 20 epochs did not improve the best validation performance, supporting epoch 20 as a suitable stopping point. A per-case before/after comparison (Dice, HD, ASSD) for few cases has been provided in the Table 12.

**Table 7:** Epoch-wise retraining metrics at key checkpoints.

Epoch	Training Loss (Dice + CE)	Dice
0	0.190	0.9322
5	0.147	0.9475
10	0.123	0.9561
15	0.126	0.9550
20	0.116	0.9586

### Ablation study: uncertainty, weights, and cycles

Table 8 summarizes Phase 1 ablations over uncertainty thresholds, weighting, and number of cycles. The best overall setting on TEST was a single cycle without weighting at *τ*_*D*_ = 0*.*92. Adding a second cycle or strong Dice-gap weighting did not improve global performance and, in our data, slightly degraded HD/ASSD, consistent with mild overfitting to a small flagged subset. We report HD (max) in pixels on 1024 × 1024 masks, and because it is a maximum surface distance metric (highly sensitive to even a single outlier), the absolute values can appear large.

**Table 8:** Ablations (Phase 1): uncertainty threshold *τ*_*D*_, weighting, and feedback cycles. Interventions = flagged clinician corrections.

*τD*	Weight	Cycles	Interv	TEST Dice	TEST HD	TEST ASSD	VAL Dice	VAL HD	VAL ASSD
0.92	none	1	104	0.9449	281.82	18.72	0.9451	264.04	18.67
0.92	none	2	208	0.9357	431.86	32.51	0.9357	459.46	34.48
0.92	dice_gap	1	104	0.9349	376.69	26.30	0.9340	395.62	28.19
0.92	dice_gap	2	208	0.9185	514.13	41.94	0.9178	532.98	45.45

### Intervention–accuracy trade-off

**Fig. 5:**
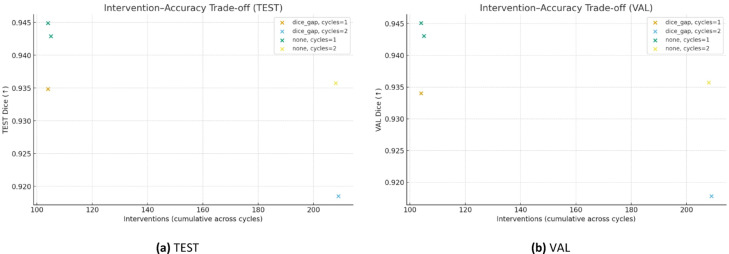
Intervention–accuracy trade-off (Dice ↑) on the held-out TEST (**a**) and validation (**b**) sets.

Figure 5 visualises the trade-off between clinician interventions and accuracy. As interventions increase (more flagged cases and cycles), Dice does not improve further on our dataset and boundary metrics (HD/ASSD) worsen, indicating diminishing returns beyond a single cycle.

### Hard-case fine-tuning (Phase 2)

We then performed a Phase 2 refinement that starts from the MedSAM base line and *trains only on the hardest flagged cases* (top 50% by lowest Dice at *τ*_*D*_ = 0*.*92). Using 5 and then 3 hardest cases across two cycles, Dice improved slightly while ASSD and VAL metrics were stable or better. Table 9 reports per-cycle outcomes.

**Table 9:** Phase 2 hard-case fine-tuning (Dice-only gate *τ*_*D*_ = 0*.*92). We retrain on the hardest flagged cases (lowest baseline Dice).

Weight	Cycles	Flags	TEST Dice	TEST HD	TEST ASSD	VAL Dice	VAL HD	VAL ASSD
none	1 (5 hard)	5	0.94728	161.37	13.97	0.94763	157.33	14.23
none	2 (5 + 3 hard)	8	0.94791	163.36	13.80	0.94809	157.44	14.16
dice_gap	1 (5 hard)	5	0.94744	161.40	13.92	0.94765	157.34	14.20

## Discussion

This section provides a comprehensive analysis of how the iterative feedback loop with clinician input enhanced the accuracy and reliability of the HybridMS model in medical image segmentation. HybridMS improves upon prior human-in-the-loop models like MedUHIP by introducing dynamic uncertainty-based clinician feedback and weighted retraining strategies, enabling more efficient and clinically aligned segmentation refinement. Furthermore, while MedSAM is a strong multi-modal segmentation model designed for diverse medical imaging tasks, it operates with a static inference architecture and lacks mechanisms for post-deployment adaptation. In contrast, HybridMS uniquely introduces adaptive correction ability, real-time learning from clinician feedback, and dynamic feedback integration during deployment. These innovations allow HybridMS to continually refine its outputs in response to clinical needs, addressing a critical gap that MedSAM does not inherently solve. It also evaluates the influence of the Dice score coefficient as a validation metric on the model’s iterative improvement. By integrating continuous learning and human expertise, this study successfully aligned the HybridMS model’s outputs with high clinical standards.

### Comparative analysis of deep learning models

**Fig. 6:**
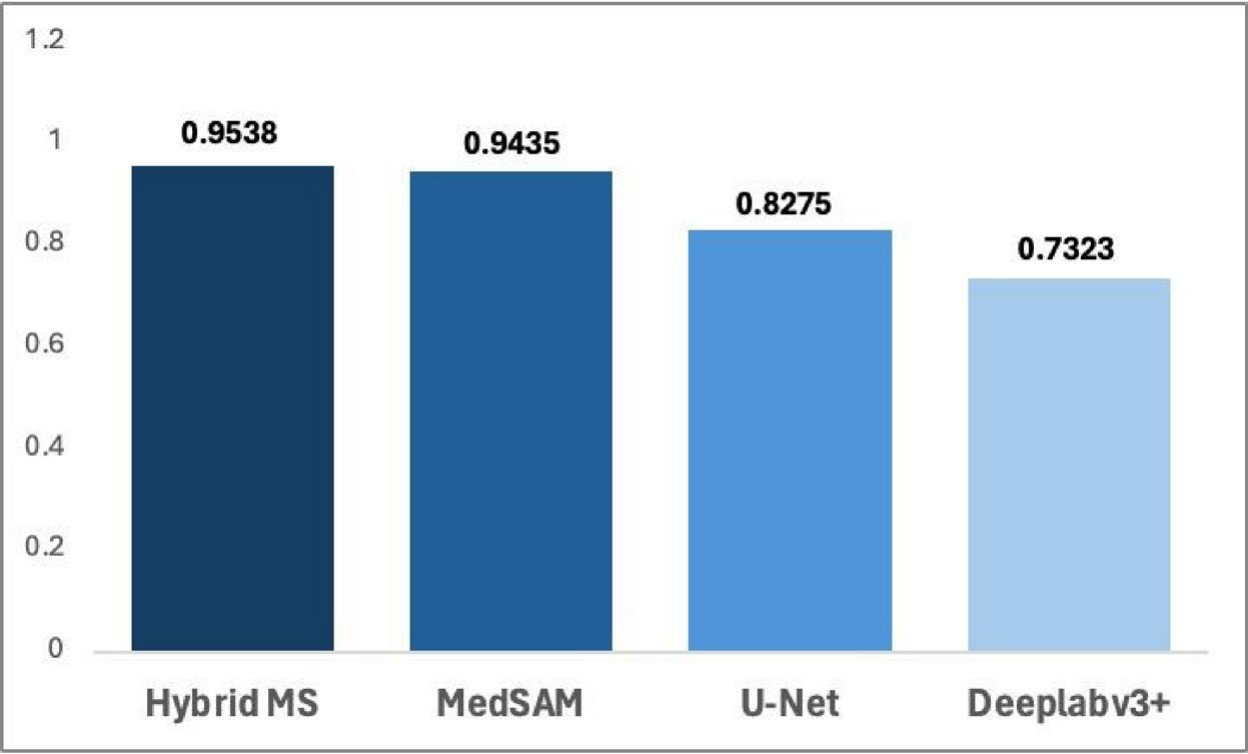
HybridMS compared to other Deep Learning models.

The performance metrics of the deep learning models MedSAM, HybridMS, U-Net, and Deeplabv3+ highlight the varying degrees of accuracy and effectiveness in segmenting lung X-rays^24^ as depicted in Fig. 6. While nn-UNet is a prominent model for medical image segmentation, we chose not to retrain it on our dataset due to the significant computational resources and time required for optimization. Additionally, nn-UNet, being a static model, may not effectively adapt to the continuous clinical variations present in our dataset, a challenge that HybridMS addresses with its adaptive learning approach. Instead, we have referenced benchmark results from studies like the LUNA16 dataset^25^, where nn-UNet demonstrated a Dice score of approximately 0.98, to provide context for comparison and highlight the expected performance of nn-UNet under more optimal conditions.

HybridMS demonstrated the best performance among the models, achieving a Dice Score of 0.9538 and an Intersection over Union (IoU) Score of 0.9126. It also exhibited strong precision (0.9539) and recall (0.9544), indicating that this hybrid approach, which integrates clinically relevant data, offers advantages in accurately delineating lung tumors.

MedSAM closely followed HybridMS, with a Dice Score of 0.9435 and an IoU Score of 0.8941. It showed high precision (0.9603) and recall (0.9288), and a balanced F1 Score of 0.9435. While effective, MedSAM lagged in achieving the clinical relevance and accuracy seen in HybridMS. U-Net, a widely recognized model in medical image segmentation^26^, performed noticeably lower than both MedSAM and HybridMS, with a Dice Score of 0.8275 and an IoU Score of 0.7081. Despite its respectable recall of 0.8919, the U-Net’s precision (0.7789) was lower, indicating potential challenges in minimizing false positives during segmentation tasks^27^. The F1 Score of 0.8275 reflects this balance between precision and recall but highlights the need for improvements in precision to match the top-performing models, Hence it cannot be relied on for medical image segmentation^28–30^.

Deeplabv3+ ^31–33^ lagged behind the other models, achieving the lowest scores across all metrics. Its Dice Score of 0.7323 and IoU Score of 0.5825 indicate relatively weaker segmentation performance. With a precision of 0.7559 and a recall of 0.7351, Deeplabv3+ shows limitations in both identifying true positives and avoiding false positives. The F1 Score of 0.7323 further confirms its comparatively lower segmentation accuracy.

**Fig. 7:**
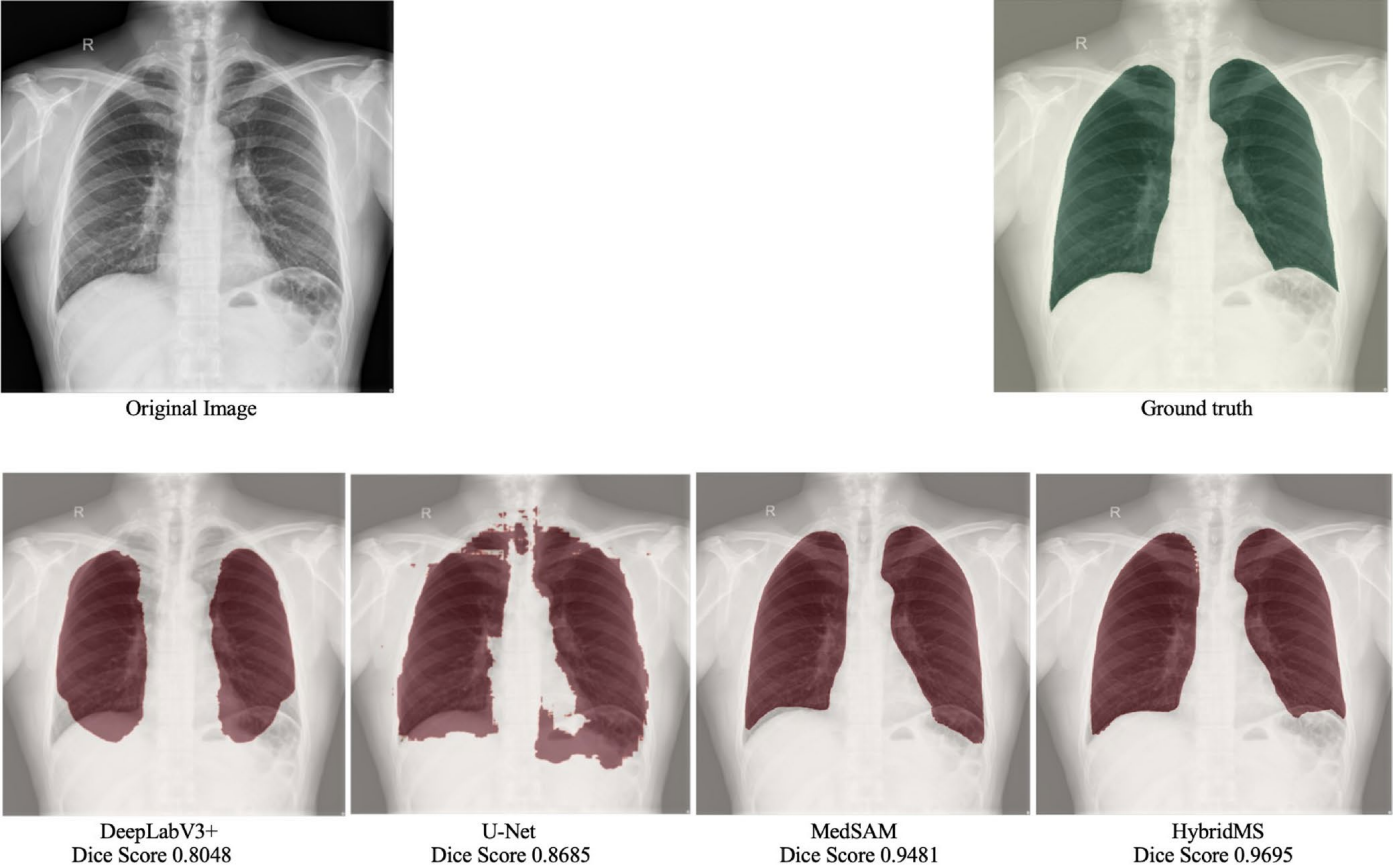
Comparison of predictions by different Deep Learning models.

In summary, HybridMS outperformed MedSAM and traditional models like U-Net^34^ and Deeplabv3+ in the segmentation of lung X-rays, with highest accuracy and clinical relevance as in Table 10. The lower performance of U-Net^35^ and Deeplabv3 + suggests that while they are effective in medical image segmentation tasks, they may require further refinement to achieve the level of accuracy demonstrated by the more advanced models like HybridMS and MedSAM as in Fig. 7.

**Table 10:** Performance comparison of baseline models and the proposed HybridMS.

Model	Dice Score	IoU Score	Precision	Recall	F1 Score
Deeplabv3+	0.7323	0.5825	0.7559	0.7351	0.7323
U-Net	0.8275	0.7081	0.7789	0.8919	0.8275
MedSAM	0.9435	0.8941	0.9603	0.9288	0.9435
HybridMS	0.9538	0.9126	0.9539	0.9544	0.9538

In this comparative analysis, U-Net, DeepLabV3 + , and MedSAM were selected to represent classical CNN architectures, semantic segmentation state-of-the-art models, and transformer-based models, respectively. This diverse selection ensures a comprehensive benchmark across major segmentation paradigms. While the overall performance improvement of HybridMS over MedSAM appears modest ( 1%), HybridMS demonstrates particularly strong improvements in challenging cases where the initial Dice score was below 0.92, achieving up to 4.8% higher segmentation accuracy. This justifies its clinical relevance, especially in difficult scenarios where precise segmentation is critical. Additionally, since the time of our experimentation, MedSAM2 has been introduced with enhanced generalization capabilities. However, our contribution in HybridMS lies not in selecting a specific backbone, but in designing a human-in-the-loop adaptive feedback mechanism. HybridMS is fundamentally model-agnostic and can be extended to work with newer architectures like MedSAM2, nnUNet, or SAM-Med2D in future implementations.

*Interpreting the ablations: *Across uncertainty thresholds, weights, and cycles, we found that a *single* feedback cycle without sample re-weighting provided the best overall balance of accuracy and boundary quality (Table 8). Additional cycles and strong Dice-gap weights increased clinician effort and tended to worsen HD/ASSD on the held-out test set, likely due to overfitting to a small, biased flagged subset. By contrast, a compact Phase 2 refinement that targets only the hardest cases yielded small but consistent improvements in Dice/ASSD (Table 9), without incurring broad intervention costs. These observations support our design choice of *selective, minimal* intervention with occasional targeted fine-tuning, aligning with HybridMS’s goal of maximizing clinical impact per unit of expert effort.

#### nnU-Net as a benchmark reference

While nnU-Net represents a leading state-of-the-art framework for medical image segmentation, retraining it on our dataset was not feasible due to its substantial computational demands and the static nature of its training pipeline. As such, we reference nnU-Net performance here in a contextual rather than a directly comparative manner. Specifically, nnU-Net achieves Dice scores of approximately 0.98 on large-scale public lung segmentation benchmarks such as LUNA16, which provides a useful reference point for understanding the upper bound of achievable performance in this domain. However, these results are not directly comparable to ours, given differences in dataset composition, imaging modality characteristics, and evaluation protocols. Our aim in including this reference is to position HybridMS performance within the broader landscape of segmentation research, rather than to claim equivalence or superiority. Future work will explore benchmarking HybridMS against nnU-Net and other high-performing architectures on common datasets to enable more direct comparisons.

Beyond nnU-Net, several recent works have introduced complementary innovations that could inform future extensions of HybridMS. For instance, *DA-TransUNet* integrates spatial and channel dual attention mechanisms with Transformer-based U-Net backbones to enhance feature representation^36^, while *FKD-Med* demonstrates privacy-preserving and communicationoptimized segmentation via federated learning and lightweight model distillation^37^. Similarly, the *Mutual Inclusion Mechanism* framework^38^ improves precise boundary segmentation through collaborative mask refinement. Incorporating such approaches into the HybridMS pipeline could further improve both segmentation performance and clinical applicability.

#### Clinical impact and future directions

We acknowledge the reviewer’s important point regarding the connection between segmentation performance improvements and actual clinical outcomes. While this study primarily focused on quantitative segmentation metrics (such as Dice coefficient, Hausdorff Distance, and ASSD), it is well recognized that improved segmentation accuracy, especially boundary precision, can directly impact clinical tasks such as surgical planning, radiotherapy targeting, and diagnosis consistency.

Although we have not yet conducted a formal clinical case study or direct manual effort reduction analysis, the improvements observed in boundary metrics (e.g., reductions in Hausdorff Distance and ASSD after feedback-driven retraining) strongly suggest a positive effect on clinical workflows. In particular, more accurate boundary delineations can significantly reduce the need for manual corrections, lower radiologist workload, and expedite diagnostic decision-making.

We recognize the importance of validating these assumptions through prospective clinical studies. As future work, we plan to perform structured clinical evaluations, involving direct measurement of manual correction times, workflow improvements, and outcome consistency before and after using HybridMS. This will provide concrete evidence of the real-world clinical benefits of our human-in-the-loop continuous learning framework.

### Significance of the Dice Score in clinical segmentation

The Dice Score Coefficient (DSC) is a crucial metric in medical image segmentation, defined as:

$$DSC = 2 \times \frac{{\left| {A \cap B} \right|}}{\left| A \right| + \left| B \right|}$$

where *A* and *B* are the sets of pixels in the predicted mask and the ground truth mask, respectively. |*A* ∩ *B*| denotes the number of pixels common to both the predicted and ground truth masks (i.e., their intersection), while |*A*| and |*B*| represent the number of pixels in the predicted and ground truth masks, respectively.

The DSC is chosen primarily for its effectiveness in measuring the overlap between predicted and ground truth masks. It balances precision and recall, making it an ideal metric for ensuring accurate and complete segmentation, which is particularly important in medical applications like lung tumor delineation. Precise segmentation is critical for treatment planning and monitoring, and the Dice Score ensures that the model accurately identifies lung regions while capturing the full extent of tumor boundaries.

A significant aspect of this study was the integration of clinician feedback into the continuous learning process of the HybridMS model. To ensure the clinical relevance of the model’s outputs, a threshold of 94% Dice Score was established. This high threshold was necessary to balance accuracy with practical clinical application. In medical imaging, particularly for tasks such as tumor segmentation, even small errors can have significant consequences. Therefore, this stringent requirement ensures that the model’s predictions are not only statistically accurate but also meet the demanding standards of clinical practice, aligning the model’s performance more closely with clinician segmentation.

### Limitations of the model

The HybridMS model, while demonstrating superior performance compared to other deep learning models, still encounters certain limitations that can affect its efficacy, particularly in real-world clinical settings. These limitations become especially apparent when dealing with edge cases, as highlighted below.

### Edge case analysis

We analysed the subset of 11 out of 107 cases (10.28%) from the test set where the MedSAM baseline achieved a Dice score of less than 0.92. These cases frequently corresponded to one or more of the following conditions:

- Poor image quality-low contrast, motion blur, or noise affecting lung boundary visibility.
- Severe anatomical variation-atypical lung shapes, deformities, or post-surgical changes.
- Presence of confounding artefacts- medical devices, rib shadows, or overlapping anatomical structures obscuring lung margins.

All 11 of these challenging cases required clinician intervention during inference to ensure clinically acceptable segmentation quality. Feedback was incorporated via the HybridMS retraining pipeline, which improved contour alignment in subsequent evaluations.

**Fig. 8:**
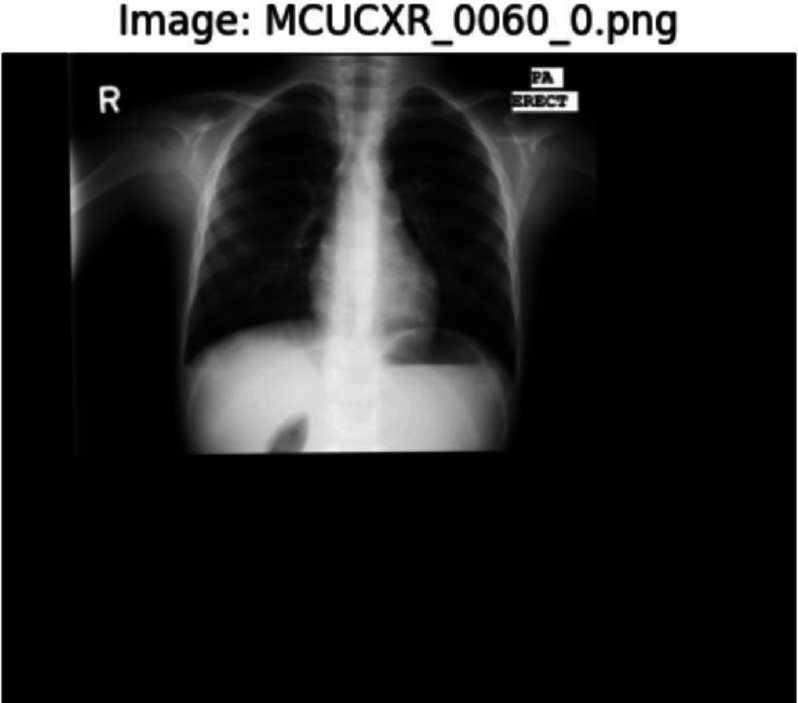
X-ray image.

**Fig. 9:**
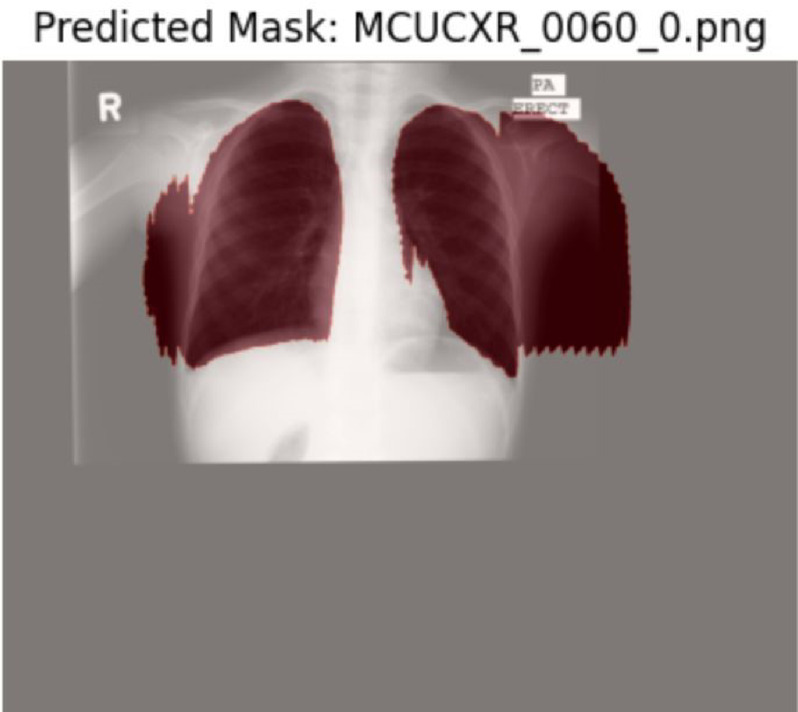
Predicted image.

Two representative examples are shown in Figs. 8 and 9. illustrating common failure modes and the corrections made. These qualitative examples demonstrate that, while HybridMS performs robustly in the majority of cases, occasional failures occur under challenging imaging conditions, underscoring the need for ongoing clinician oversight in clinical deployment.

### Data quality

The performance of the HybridMS model is heavily reliant on the quality and diversity of the datasets used during training. While the underlying MedSAM model was pre-trained on a large and diverse set of medical images providing a strong and generalised starting point the fine-tuning process in this study relied on the Shenzhen and Montgomery datasets, both of which are relatively small and originate from similar geographic regions. This limited diversity may constrain generalisability to other populations, imaging conditions, or acquisition protocols. In clinical settings, variability in image quality, patient demographics, and imaging techniques can lead to inconsistent segmentation results.

### Generalizability

While HybridMS performed well on the validation dataset, its ability to generalise to real-world, variable data remains a concern. The model may struggle with complex cases that deviate from the patterns seen during fine-tuning. The edge case illustrates how the model’s segmentation deviates from the clinician-annotated mask, indicating potential difficulties in adapting to unseen data with differing characteristics. Future work will include validation on larger, more diverse datasets such as NIH ChestX-ray14 and JSRT to assess robustness across varied clinical scenarios^39^.

### Clinical integration

Integrating HybridMS into clinical workflows can be complex and time-consuming. While these preliminary findings demonstrate substantial efficiency gains, they are based on a small sample size and a limited number of annotators. A larger-scale, structured clinical validation study is proposed to further substantiate these results and evaluate performance across a broader range of radiologists and imaging conditions. As shown in the edge case, even with continuous learning mechanisms, discrepancies between the model’s predictions and clinician-provided ground truth may still occur, necessitating further refinement.

### Ethical concerns

Ensuring data privacy, anonymisation, and adherence to ethical guidelines is crucial. In scenarios where the model’s output could influence clinical decisions, it is imperative to maintain transparency and allow for human oversight to mitigate potential risks.

### Future work

Future work will focus on several key areas to build upon the findings of this research. Enhancing the model’s generalizability will be a primary objective, aiming to improve its performance across diverse patient populations and imaging conditions by expanding the training dataset and employing advanced data augmentation techniques. Additionally, integrating multimodal data, such as combining chest X-rays with CT scans or MRI, will be explored to further improve segmentation accuracy. Real-time clinical integration of the HybridMS model will be another critical area, involving rigorous testing in clinical settings to ensure that the model meets the practical needs of clinicians. Addressing ethical concerns will also be a priority, particularly in relation to data privacy, patient consent, and the transparency of AI-driven decisions. Finally, research will explore ways to enhance the model’s continuous learning capabilities, allowing it to quickly adapt to new data and feedback, thereby maintaining its clinical relevance over time.

## Conclusion

This dissertation has demonstrated the potential of hybrid intelligence, particularly through integrating the MedSAM model with continuous clinician feedback resulting in the development of HybridMS, to significantly enhance the accuracy and clinical relevance of medical image segmentation. Focusing on lung tumor segmentation in chest X-rays-a critical area in diagnostics, This research illustrates how combining advanced machine learning techniques and human expertise can lead to improved outcomes.

The HybridMS model, powered by the Vision Transformer (ViT) architecture, initially showed strong performance in segmenting lung regions. Its accuracy was further improved through an iterative feedback loop where clinician-corrected masks were used to retrain the model. This approach led to a noticeable increase in key performance metrics, such as Dice Score and Intersection over Union (IoU), highlighting the benefits of continuous learning in refining AI models.

The study emphasized the crucial role of clinician feedback in ensuring the model’s outputs meet high clinical standards. By setting a stringent threshold of 94% for the Dice Score, the research aligned the model’s performance with the rigorous demands of clinical practice, enabling it to adapt and improve in real-time. In addition to accuracy, the HybridMS model significantly increased efficiency by reducing the time required for manual annotation by 82%. This not only speeds up diagnostics but also reduces clinicians’ cognitive load, allowing them to focus on more complex cases.

The research identified limitations in handling edge cases and generalizability across diverse datasets, emphasizing the need for ongoing refinement and a hybrid AI-human approach. It also highlighted the importance of ensuring AI models like HybridMS are both technically robust and clinically relevant, aligning outputs with clinical standards and addressing ethical concerns like data privacy to enhance precision and reliability in medical diagnostics.

### Theoretical foundation for hybrid intelligence

Hybrid Intelligence (HI) refers to the synergistic combination of human and machine intelligence, aiming to overcome the individual limitations of each through close collaboration. As outlined by Dellermann et al. (2019), HI systems foster dynamic co-adaptation between humans and AI agents, where both entities learn and improve over time. Holzinger et al. (2016) emphasized that in domains characterized by uncertainty, such as healthcare, human expertise remains irreplaceable and should be augmented rather than replaced by AI.

Kamar (2016) identified that successful Hybrid Intelligence designs prioritize selective human involvement based on system uncertainty, emphasizing the strategic delegation of tasks between human experts and algorithms. In line with this, Dellermann et al. (2021) provided key design guidelines for Hybrid Intelligence systems, highlighting the importance of feedback loops, uncertainty handling, and human-centric learning. In the medical domain, Wang et al. (2022) demonstrated that dynamically incorporating clinician expertise during AI training enhances the robustness and trustworthiness of the model.

Reflecting these principles, HybridMS operationalizes Hybrid Intelligence by employing uncertainty-driven dynamic feedback, where the model continuously monitors its prediction confidence and requests clinician input only when confidence falls below a predefined threshold, thereby optimizing clinician effort. Furthermore, HybridMS uses a weighted retraining strategy, giving greater importance to critical clinical corrections made by experts during retraining to ensure that improvements target clinically significant segmentation errors. Through continuous co-adaptive learning, HybridMS iteratively refines its segmentation capabilities, aligning its outputs with clinician standards and clinical expectations over time.

Thus, HybridMS is not merely an AI model augmented with human corrections but a co-evolving Hybrid Intelligence system designed to dynamically adapt, learn, and improve in response to human expertise within clinical workflows.

## Methods

### Data collection and preparation

The dataset used in this study was specifically curated to address the critical need for accurate segmentation of lung tuberculosis in chest X-rays, a condition closely associated with lung cancer. Two primary datasets were utilized: the China Set—Shenzhen Chest X-ray Database and the Montgomery County Chest X-ray Database. The Shenzhen dataset comprised 336 cases with manifestations of tuberculosis and 326 normal cases, with images in PNG format at approximately 3 K x 3 K pixels. The Montgomery dataset, developed in collaboration with the Department of Health and Human Services, Montgomery County, Maryland, USA, contained 58 cases of tuberculosis and 80 normal cases, also in PNG format, but with a resolution of 4020 × 4892 pixels. These datasets provided a robust foundation for training and validating the HybridMS model, with lung boundaries meticulously segmented under the supervision of radiologists.

In preparing the data for model training, several critical preprocessing steps were undertaken. Initially, clinician-segmented masks, originally in .nrrd format, were converted to .png format to ensure compatibility with the model’s input requirements. The manual segmentation work was done using Slicer 3D under the supervision of clinician of B. Borooah Cancer Institute who helped in creating a high-quality, clinically relevant training dataset. The dataset was divided into 3 subset: Train, Test and Validation with a proportions of 70%, 15% and 15%, respectively to ensure that model is properly evaluated and generalized.

To ensure consistency and ease of access during the training process, the segmented masks were systematically organized and renamed. Additionally, to enhance the model’s robustness and prevent overfitting, data augmentation techniques such as random rotations, flips, and color adjustments were applied, effectively expanding the dataset and improving the model’s ability to generalize across diverse cases^40^. Image normalization was performed to standardize intensity values across the dataset, ensuring uniformity and reducing potential biases. Furthermore, positional embeddings were adjusted using data interpolation, aligning the spatial dimensions of input images with the model’s expected input size, thereby ensuring accurate feature extraction.

### HybridMS model architecture

**Fig. 10:**
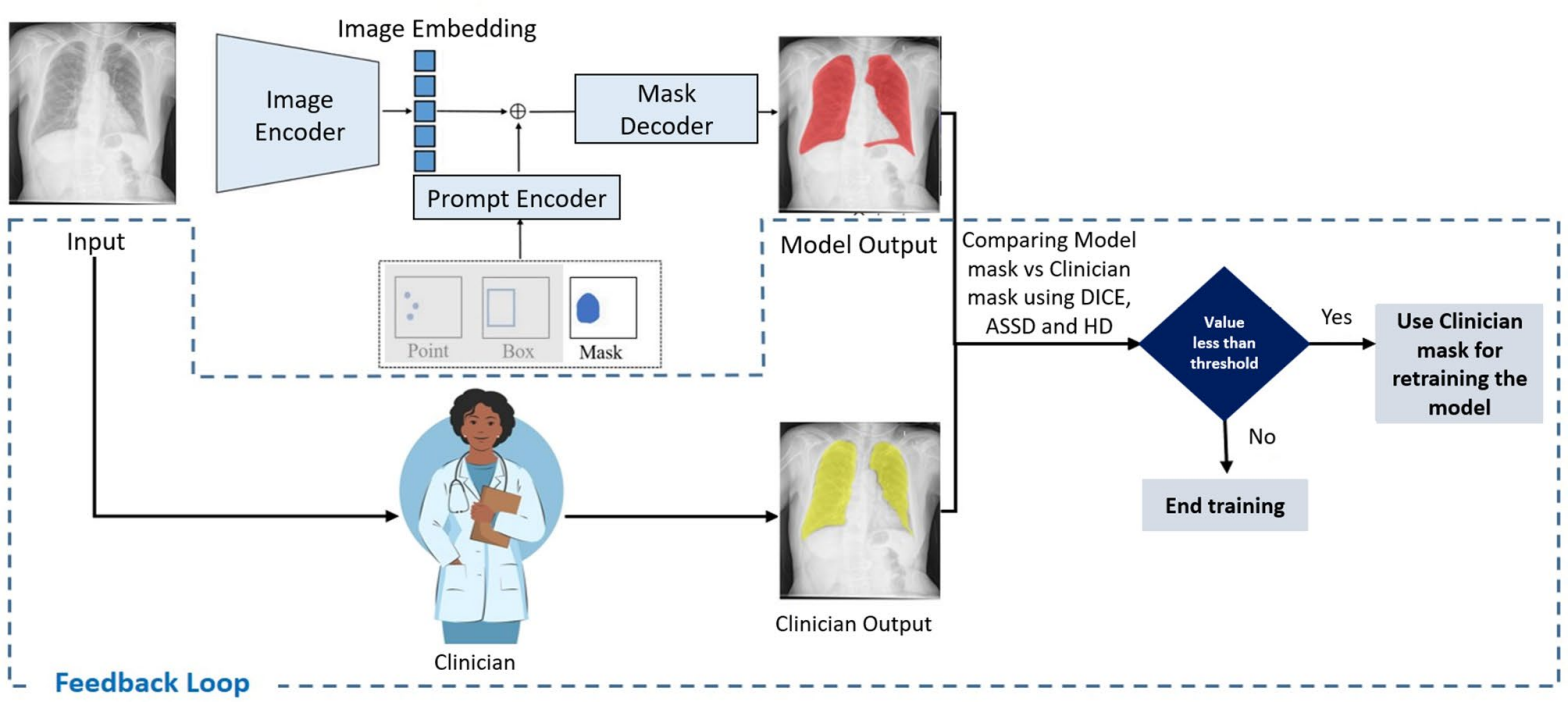
System Architecture of the proposed HybridMS model integrated with Continuous Learning. The diagram illustrates the process flow, starting from input image decoding to segmentation mask generation, comparison with clinician output, and the iterative feedback loop for model retraining.

The architecture of HybridMS is designed to leverage modern deep learning techniques while incorporating real-time feedback from clinicians, ensuring alignment with clinical standards. Figure 10. The key components of the architecture:

#### Vision Transformer (ViT) architecture

At the core of HybridMS is the Vision Transformer (ViT), which acts as the image encoder. The ViT processes high-resolution chest X-ray images by dividing them into sequences of flattened 2D patches. These patches are then fed into a series of 12 transformer layers, each composed of multi-head self-attention blocks and Multilayer Perceptron (MLP) blocks. This structure captures complex relationships between different regions of the image, making it particularly effective for segmentation tasks. The final image embedding produced by the ViT is downscaled to 64 × 64 pixels, 16 times smaller than the original image size, enabling efficient processing while preserving critical spatial information.

#### Prompt encoder

The prompt encoder integrates user-provided inputs (e.g., bounding boxes, points, or masks) into the segmentation process. In the initial round (before clinician feedback), HybridMS follows the standard MedSAM prompting workflow: bounding box prompts are generated from connected components of the baseline predicted mask, with a fixed ± 5 pixel margin to ensure complete coverage of the predicted lung fields. No random spatial shifts are applied. These bounding boxes are then encoded using positional encodings combined with learned embeddings. No point prompts (positive or negative) or initial mask prompts are used in the first round.

The encoded prompts are merged with the image embedding produced by the ViT, guiding the segmentation process to focus on the relevant lung regions. The resulting segmentation is then evaluated against predefined quality thresholds (Dice < 0*.*94, HD > 15 mm, or ASSD > 20 mm). If the prediction fails to meet any of these criteria, the case is flagged for retraining with clinician-corrected masks in subsequent iterations.

#### Continuous learning and adaptability

A defining feature of HybridMS is its continuous learning mechanism, enabling iterative improvements through clinician feedback. Segmentation masks generated by the model are evaluated against clinician-annotated masks using Dice coefficient, Hausdorff Distance (HD), and Average Symmetric Distance (ASSD). A Dice threshold of 0.94, HD of 15 mm, and ASSD of 20 mm are applied to flag discrepancies. HD and ASSD were specifically used to capture fine boundary errors beyond what Dice alone detects. Masks failing these criteria are incorporated into retraining, ensuring progressive refinement and high clinical reliability.

### Model training

The training process for HybridMS was meticulously designed to balance effective learning with model stability. The model was initialized using a pre-trained Vision Transformer (ViT) model, which provided a robust starting point, and was then fine-tuned using the curated chest X-ray dataset. During training, the ViT architecture was employed to extract detailed features from the chest X-ray images, forming the foundation for accurate segmentation. The model was trained to generate segmentation masks that delineate tuberculosis regions in the lung images, with the accuracy of these masks being critical to the model’s overall performance in clinical settings.

Training of the model was conducted using the training dataset. To optimize the training process, a combination of Dice loss and cross-entropy loss was employed as the loss function. Dice loss was used to maximize the overlap between predicted and actual masks, ensuring high accuracy in the segmentation output^41^, while cross-entropy loss minimized pixel classification errors, further refining the model’s predictions.

The hyperparameters were finely tuned to ensure that the learning process was both effective and stable. A learning rate of 0.0001 was selected to balance rapid convergence with the need to avoid overshooting the optimal model weights. A weight decay of 0.01 was applied to prevent overfitting and to enhance the model’s generalization capabilities across different datasets. The batch size was carefully chosen to accommodate the high-resolution images in GPU memory, and the model was trained over 20 epochs, which was determined to be optimal for avoiding overfitting while ensuring comprehensive learning from the dataset. Several data augmentations techniques were applied to make the data better fit for training which was earlier explained in data collection and preparation step.

### Validation and performance assessment

The performance of HybridMS was rigorously evaluated using a separate validation dataset, comprising lung X-ray images that were not included in the training set. This validation process was crucial for assessing the model’s ability to generalize to new data and produce clinically relevant segmentation results. The Dice score coefficient was used as the primary metric to measure the accuracy of the generated segmentation masks, providing an objective and quantifiable measure of the model’s performance. Discrepancies between the model’s predictions and the ground truth were carefully analyzed, identifying areas for improvement that informed subsequent iterations of model refinement.

To complement the single-split validation described above and quantify statistical robustness, we computed nonparametric bootstrap confidence intervals (B = 5000 resamples) for all metrics on the held-out test set. We report mean ± standard deviation (SD) and 95% bootstrap confidence intervals (CIs) in Table 2 of the Results section. In addition, we repeated inference five times on the same split to estimate run-to-run variability due to stochastic kernels in the model implementation, reported in the supplementary material Table .

Furthermore, to provide stronger statistical evidence of robustness, we performed a full fivefold cross-validation over stratified splits of the dataset. The results, summarised in Table 11, show mean ± SD values for Dice, IoU, Hausdorff Distance, and ASSD across folds.

**Table 11:** Five-fold cross-validation results for HybridMS. Metrics are reported as mean ± standard deviation across folds.

Metric	Mean ± Std. Dev.
Dice Score	0.9523 ± 0.0048
IoU	0.9112 ± 0.0062
Hausdorff (mm)	31.84 ± 2.10
ASSD (mm)	4.92 ± 0.23

#### Computational requirements and runtime performance

All experiments were conducted on a workstation equipped with an NVIDIA GPU (16 GB VRAM) and an Intel Xeon CPU. HybridMS achieved a mean inference time of approximately 5.2 s per case when running on the GPU, including image loading, preprocessing, and mask generation. This runtime is suitable for real-time or near-real-time clinical integration.

While HybridMS introduces an additional clinician interaction step, this is selectively triggered only for cases failing predefined quality thresholds (Dice < 0*.*94, HD > 15 mm, ASSD > 20 mm), which accounted for approximately 10% of the test cases in this study. This targeted intervention strategy minimises disruption to the clinical workflow while ensuring that problematic cases receive focused attention. The majority of cases can be processed fully automatically, enabling substantial time savings without sacrificing segmentation accuracy.

#### Timing study protocol

To evaluate workflow efficiency in a manner consistent with clinical practice, we conducted a preliminary timing study with two board-certified radiologists experienced in thoracic imaging. The study measured the time required to produce final, clinically acceptable lung segmentation masks under two conditions: (i) fully manual segmentation and (ii) HybridMS-assisted segmentation.

Timing was recorded using a standardized stopwatch protocol, starting from the moment the image was loaded into the annotation software and ending when the segmentation was approved as final by the annotator. For HybridMS-assisted cases, the pre-generated segmentation from HybridMS was loaded first, after which the radiologist could make manual refinements if necessary before approval.

To ensure comparability, the same workstation, display settings, and annotation software were used for all measurements, and cases were presented in randomized order. A total of 20 cases were timed: 10 “standard” cases (MedSAM Dice ≥ 0*.*92) and 10 “difficult” cases (MedSAM Dice < 0*.*92), as defined in Sect. “Inference time and computational considerations”. The results of this timing study, reported in the Results section, provide preliminary evidence of HybridMS’s potential to substantially reduce annotation time in both standard and challenging scenarios. While the sample size is modest, these findings offer an initial estimate of workflow gains and will be validated in a larger-scale clinical study in future work.

### Model retraining with incorporation of clinician feedback-hybrid intelligence

A cornerstone of the HybridMS model is its hybrid intelligence framework, which integrates continuous learning with real-time clinician feedback to maintain alignment with clinical standards and adapt to evolving medical practices. This approach is designed to systematically enhance the model’s accuracy, robustness, and clinical relevance over time, establishing HybridMS as a reliable tool for critical diagnostic tasks.

The continuous learning process initiates with HybridMS generating segmentation masks based on input images and prompts. These generated masks are rigorously compared against manually segmented clinician-provided masks using multiple evaluation metrics: Dice similarity coefficient, Hausdorff Distance (HD), and Average Symmetric Distance (ASSD). While Dice assesses overall volumetric overlap, HD measures the largest boundary deviation, and ASSD quantifies the mean boundary error, providing a more sensitive assessment of segmentation quality. Specific thresholds were defined: a Dice score below 0.94, a Hausdorff Distance exceeding 15 mm, or an ASSD above 20 mm triggers the mask to be flagged for retraining. These thresholds ensure that both gross segmentation mismatches and subtle boundary inaccuracies are detected.

Whenever a predicted mask fails to meet any of the defined criteria, the corresponding clinician-corrected mask is incorporated into the retraining set. This feedback loop is repeated iteratively, with each cycle refining the model’s segmentation performance and enhancing its ability to generalize to new and clinically diverse data. Through this mechanism, HybridMS does not merely correct its previous mistakes but actively learns from them, strengthening its decision boundaries and improving boundary precision.

By combining expert-driven annotations with state-of-the-art machine learning strategies and multi-metric evaluation, the HybridMS framework ensures that the model remains not only accurate but also dynamically adaptable to clinical expectations. This methodical retraining strategy positions HybridMS as an advanced, clinician-aligned system for high-stakes medical image analysis.

**Terminology: Continuous vs. Continual Learning** In this study, we use the term *continuous learning* to refer to iterative updates performed within the same deployment cycle, where model parameters are refined in near real-time through an active feedback loop with clinicians. This is distinct from *continual learning*, which generally describes long-term adaptation of a model across successive tasks or datasets over extended periods. Continuous learning in HybridMS is designed for immediate integration of corrections into the current workflow, whereas continual learning would focus on evolving the model over broader, multi-task scenarios.

#### Uncertainty quantification and weighted retraining strategy

A key innovation of HybridMS is its selective feedback triggering mechanism, designed to request clinician input only when the model’s prediction confidence is low or when segmentation quality falls below clinically relevant thresholds. Confidence estimation is implemented via a hybrid metric-based uncertainty quantification: for each segmentation mask, Dice similarity coefficient (DSC), Hausdorff Distance (HD), and Average Symmetric Surface Distance (ASSD) are computed against the corresponding baseline (MedSAM) prediction. If the DSC falls below 0.94, HD exceeds 15 mm, or ASSD exceeds 20 mm, the case is flagged as “uncertain” and sent for clinician review.

This metric-driven gating acts as a proxy for predictive uncertainty, ensuring that clinician effort is reserved for the most challenging or error-prone cases. While more sophisticated uncertainty estimation techniques such as Monte Carlo dropout or entropy-based variance could be integrated in future iterations, the current approach prioritizes computational efficiency and practical deployment.

For retraining, clinician-corrected masks are incorporated into the training set with weighted importance to accelerate learning from high-value corrections. Weights are assigned on a continuous scale proportional to the severity of the discrepancy between the model’s initial prediction and the clinician-corrected mask. Specifically, the weighting factor *w* is defined as:

$$w = 1 + \alpha \cdot (1 - DSC),$$

where *α* is a scaling parameter (set to 2 in this study). This formulation ensures that masks with larger segmentation errors contribute more strongly to gradient updates, reinforcing learning on clinically significant corrections while maintaining stability across the training process.

By combining uncertainty-based selection with weighted retraining, HybridMS minimizes unnecessary intervention, focuses learning on the most informative cases, and maintains high clinical relevance while keeping computational demands manageable.

#### Ablation protocol

We systematically ablated four design axes of HybridMS:

(i) ***Uncertainty thresholds*** We tested the main tri-metric gate (Dice/HD/ASSD = (0*.*92*,*60 mm*,*10 mm)) and a Dice-only gate (Dice = 0*.*92, HD/ASSD disabled) to probe sensitivity to boundary metrics.
(ii) ***Retraining weights*** Decoder/prompt updates used either no weighting or a DSC-gap weight, w=1+α (1−DSC), *α* = 2 for the main grid*,* applied per image during loss aggregation.
(iii) ***Number of feedback cycles*** We ran one vs two clinician-in-the-loop cycles. Each cycle freezes the image encoder and trains the prompt encoder + mask decoder for 10 epochs (Dice + BCE losses), continuing from the prior cycle’s weights.
(iv) ***Prompt strategy*** Retraining used the clinically realistic *mask-prompt* (clinician mask downsampled to 256 × 256) as the fixed prompting mode; evaluation used GT-derived bounding boxes to prompt the decoder. No other prompts were tried as the base model MedSAM works with mask prompt.

For every setting we report the number of *interventions* (flagged cases) and plot intervention–accuracy trade-offs on VAL and TEST (Dice ↑, HD ↓, ASSD ↓).

### Iterative improvement and final evaluation

The feedbackretraining cycle was performed once for each image in this study. After initial inference with bounding box prompts  (see Sect “Prompt Encoder”), cases failing the predefined quality thresholds (Dice < 0*.*94, HD > 15 mm, ASSD > 20 mm) were corrected by clinicians and incorporated into a single retraining iteration.

This process ensured that the most clinically significant errors were addressed without excessive retraining overhead. With the updated model, performance metrics such as Dice score, precision, recall, and F1 score were recalculated for the test set. A before-and-after comparison of Dice, HD, and ASSD for few retrained cases is presented in the Table 12. This can also be visualised from the segmented images attached in Fig. 3. showing consistent improvements, especially in challenging scenarios such as low-contrast or anatomically atypical chest X-rays.

**Table 12:** Representative examples of per-case improvement after clinician-guided retraining (MedSAM → HybridMS). ∆ denotes after → before. These examples illustrate the pattern observed in improved cases.

Case	Dice_b_	Dice_a_	∆Dice	HD_b_ (mm)	HD_a_ (mm)	∆HD	ASSD_b_ (mm)	ASSD_a_ (mm)	∆ASSD
84	0.8422	0.8500	+ 0.0078	408.08	362.50	− 45*.*58	94.85	87.37	− 7*.*48
21	0.7122	0.7181	+ 0.0059	697.17	679.15	− 18*.*02	144.32	140.72	− 3*.*60
74	0.8737	0.8782	+ 0.0046	501.81	455.36	− 46*.*45	78.17	73.35	− 4*.*82
6	0.9266	0.9304	+ 0.0037	150.88	84.91	− 65*.*97	18.39	15.14	− 3*.*26
85	0.9480	0.9499	+ 0.0019	125.40	124.43	− 0*.*97	29.62	28.64	− 0*.*98

While additional feedback–retraining cycles could be performed, we found that a single iteration was sufficient to achieve the majority of measurable improvement, balancing accuracy gains with computational efficiency. To quantitatively assess segmentation quality, we report Dice similarity coefficient (DSC) for volumetric overlap and normalized surface distance (NSD) for boundary agreement, ensuring that both global accuracy and fine boundary alignment were evaluated.

## Comparison of HybridMS and MedSAM

To clarify the distinctions between HybridMS and the MedSAM baseline, we provide a side-by-side comparison across architecture, training strategy, and clinical integration as shown in the Table 13 below.

**Table 13:** Key differences between HybridMS and MedSAM.

Aspect	MedSAM (baseline)	HybridMS (proposed)
Architecture backbone	Vision Transformer (ViT-B) with prompt encoder and mask decoder	Same ViT-B backbone and prompt encoder as MedSAM, but integrated into a continuous learning framework with clinician feedback
Prompt usage	Initial mask prompt from bounding box; no iterative refinement	Initial mask prompt from bounding box, followed by clinician-corrected masks fed back into retraining
Training strategy	Single-pass training on curated dataset	Hybrid intelligence loop: postprediction evaluation (Dice, HD, ASSD), clinician feedback, and selective weighted retraining
Clinical feedback integration	None	Integrated feedback after cases failing predefined thresholds (Dice < 0.94, HD > 15 mm, ASSD > 20 mm)
Adaptability to difficult cases	(Limited; fixed model weights after initial training	Improves over time by learning from challenging cases, refining decision boundaries and contour precision
Workflow efficiency	No explicit timing optimisation	Demonstrated ∼82% time reduction for standard cases and ∼60% for difficult cases in preliminary radiologist tests

This comparison highlights that while HybridMS leverages the same architectural backbone as MedSAM, its iterative clinician-in-the-loop design enables progressive performance gains, particularly in challenging segmentation scenarios, while also improving clinical workflow efficiency (Table 14).

**Table 14:** Per-fold cross-validation results for HybridMS. Metrics are reported per fold.

Fold	Dice	IoU	Hausdorff (mm)	ASSD (mm)
1	0.9495	0.9080	33.12	5.10
2	0.9551	0.9138	31.02	4.88
3	0.9518	0.9105	32.45	4.91
4	0.9560	0.9149	30.77	4.75
5	0.9491	0.9088	32.84	4.96

## Data Availability

The datasets generated and/or analysed during the current study are available in the [Zenodo] repository, 10.5281/zenodo.14059287. Link: https://zenodo.org/records/14059288
